# Hypoparathyroidism induced by immune checkpoint inhibitors: a review of literature reports and real-world pharmacovigilance data

**DOI:** 10.1530/EC-25-0123

**Published:** 2025-07-12

**Authors:** Jiaxun Jiao, Zongyun Li, Xiaoli Zhu, Linwei Chen, Yuting Wu

**Affiliations:** ^1^ Department of Spinal Surgery, The People’s Hospital of Hengshui, Hengshui, Hebei, China; ^2^ Department of Pharmacy, The People’s Hospital of Hengshui, Hengshui, Hebei, China; ^3^ Department of Pharmacy, The Affiliated Taizhou People’s Hospital of Nanjing Medical University, Taizhou, Jiangsu, China

**Keywords:** immune checkpoint inhibitors, hypoparathyroidism, FDA adverse event reporting system, case reports

## Abstract

**Objective:**

This study aims to comprehensively assess the characteristics of immune checkpoint inhibitor (ICI)-induced hypoparathyroidism (HP) by analyzing published case reports and adverse event data from the U.S. Food and Drug Administration Adverse Event Reporting System (FAERS).

**Methods:**

We identified case reports of HP during ICI treatment through a systematic literature search and extracted clinical data. FAERS data (Q2 2011–Q3 2024) were analyzed for cases where ICIs were the primary suspected cause of HP. Disproportionality and Bayesian analyses were used to evaluate associations between ICI classes and HP.

**Results:**

Nine ICI-related HP cases were documented in the literature. Predominant manifestations included neuromuscular symptoms (e.g., weakness, paresthesia). Laboratory findings consistently revealed hypocalcemia and low parathyroid hormone (PTH) levels. Four cases exhibited prolonged QT intervals, and calcium-sensing receptor (CaSR) autoantibodies were detected in four patients. Pharmacovigilance analysis showed the strongest signal for CTLA-4/PD-1 inhibitor combination therapy (ROR (95% CI): 6.04 (2.26–16.13); PRR (*χ*^2^): 6.04 (16.71); EBGM (EBGM05): 6.01 (2.64); IC (IC025): 2.59 (1.29)).

**Conclusion:**

ICI-induced HP is a rare endocrine toxicity requiring heightened clinical vigilance. Regular monitoring of serum calcium levels is essential, with PTH measurements recommended in the presence of hypocalcemia to facilitate early diagnosis and appropriate management.

## Introduction

Over the past decade, immune checkpoint inhibitors (ICIs) have emerged as first-line treatments for various cancers, significantly improving patient survival rates. The U.S. Food and Drug Administration (FDA) has approved three major classes of ICIs: programmed cell death receptor-1 (PD-1) inhibitors (e.g., cemiplimab, nivolumab, pembrolizumab), programmed death-ligand 1 (PD-L1) inhibitors (e.g., atezolizumab, avelumab, durvalumab), and cytotoxic T-lymphocyte-associated protein 4 (CTLA-4) inhibitors (e.g., ipilimumab). These agents enhance antitumor immunity by blocking inhibitory signals (CTLA-4 or PD-1/PD-L1 pathways), thereby restoring T-cell activity against malignant cells.

However, this immune activation can trigger immune-related adverse events (irAEs), most commonly affecting the skin, gastrointestinal tract, and endocrine system (1, 2). While endocrine toxicities such as thyroid dysfunction and hypophysitis are well documented, ICI-induced hypoparathyroidism (HP) remains exceptionally rare (3). To date, only isolated case reports from clinical trials have described this phenomenon, highlighting the need for post-marketing surveillance data to characterize its clinical profile.

This study investigates the association between ICIs and HP by analyzing real-world data from the FDA Adverse Event Reporting System (FAERS) and published case reports. We aim to elucidate the incidence patterns, clinical characteristics, and pharmacovigilance signals of this toxicity to inform clinical monitoring strategies.

## Methods

### Case series

We conducted a systematic literature review following PRISMA guidelines to identify published cases of ICI-induced HP. Databases searched included PubMed, Scopus, Web of Science, and Embase, from inception to November 2024, using terms combining generic/brand names of all seven ICIs (e.g., nivolumab, ipilimumab, Keytruda) with hypoparathyroidism, hypocalcemia, or parathyroid hormone deficiency. However, only English-language publications were included. Grey literature (e.g., conference abstracts) and non-indexed journals were excluded. Cases without confirmatory laboratory data (e.g., low PTH + hypocalcemia) were excluded, potentially omitting milder presentations. Patient demographics (sex and age), indications for treatment, involved drugs, onset time, clinical manifestations, laboratory results, treatments, and clinical outcomes were collected and analyzed.

### Disproportionality analysis

We obtained pharmacovigilance data from FAERS, a spontaneous surveillance database for post-marketing drug safety monitoring, extracting records from Q2 2011 (marking the first ICI approval by FDA in March 2011) through Q3 2024. The raw ASCII-format data underwent rigorous cleaning and deduplication procedures using R programming, with subsequent identification of relevant cases through systematic queries incorporating both generic and brand names of seven ICIs (Table 1) alongside standardized MedDRA preferred terms (‘hypoparathyroidism’ and ‘blood parathyroid hormone decreased’). Our analysis specifically retained cases where ICIs were designated as the primary suspected causative agents for hypoparathyroidism, while systematically excluding erroneous records (including instances with illogical temporal sequences such as HP onset preceding ICI administration). As this study exclusively utilized anonymized, publicly available regulatory data, institutional review board approval was unnecessary.

**Table 1:** Name of immune checkpoint inhibitor.

Generic names	Product names	Target for drugs	Year
Atezolizumab	Tecentriq	PD-L1	2016
Avelumab	Bavencio	PD-L1	2017
Durvalumab	Imfinzi	PD-L1	2017
Nivolumab	Opdivo	PD-1	2014
Pembrolizumab	Keytruda	PD-1	2014
Cemiplimab	Libtayo	PD-1	2018
Ipilimumab	Yervoy	CTLA-4	2011

CTLA-4, cytotoxic T-lymphocyte-associated protein 4; PD-1, programmed cell death protein 1; PD-L1, programmed death-ligand 1.

The demographic and clinical characteristics of the reported cases are presented in Table 2. The cohort had a median age of 60.8 years (range: 29–79 years), with comparable age distributions observed among different ICI treatment groups. Male patients predominated, comprising 72% of cases. Most reports (92%) were physician-submitted, with Japan (40%) and the United States (20%) representing the most frequent reporting countries. Clinical outcomes analysis revealed concurrent adverse events in 36% of cases, while 36% required hospitalization, and 8% resulted in life-threatening complications or fatal outcomes.

**Table 2:** Clinical characteristics of patients with hypoparathyroidism caused by ICIs from the FAERS database.

Variable	PD-1 (*n* = 15)	PD-L1 (*n* = 6)	CTLA-4 + PD-1 (*n* = 4)	Total (*n* = 25)
Sex				
Female	2 (13.3%)	2 (33.3%)	-	4 (16.0%)
Male	12 (80.0%)	2 (33.3%)	4 (100%)	18 (72.0%)
Missing	1 (6.7%)	2 (33.3%)	-	3 (12.0%)
Age (range)	64 (29–79)	60.7 (60.6–60.8)	53 (53–65)	60.8 (29–79)
Reporter				
Healthcare profession	13 (86.7%)	6 (100%)	4 (100%)	23 (92.0%)
Consumer	2 (13.3%)	-	-	2 (8.0%)
Country				
Japan	5 (33.3%)	3 (50%)	2 (50%)	10 (40.0%)
United States	5 (33.3%)	-	-	5 (20.0%)
France	2 (13.3%)	-	-	2 (8.0%)
South Korea	-	2 (33.3%)	-	2 (8.0%)
China	1 (6.7%)	1 (16.7%)	-	2 (8.0%)
Switzerland	-	-	2 (50%)	2 (8.0%)
Others	2 (13.3%)	-	-	2 (8.0%)
Outcome				
Death	2 (13.3%)	-	-	2 (8.0%)
Life-threatening	1 (6.7%)	1 (16.7%)	-	2 (8.0%)
Hospitalization	6 (40.0%)	1 (16.7%)	2 (50.0%)	9 (36.0%)
Others	4 (26.7%)	3 (50.0%)	2 (50.0%)	9 (36.0%)
Missing	2 (13.3%)	1 (16.7%)	-	3 (12.0%)
Disease				
Lung cancer	5 (33.3%)	3 (50.0%)	1 (25.0%)	9 (36.0%)
Melanoma	-	-	3 (75.0%)	3 (12.0%)
Others	6 (40.0%)	2 (33.3%)	-	8 (32.0%)
Missing	4 (26.7%)	1 (16.7%)	-	5 (20.0%)
Time to onset median (range), days	50 (1–292)	135 (43–305)	89 (37–150)	64 (1–305)

CTLA-4, cytotoxic T-lymphocyte-associated protein 4; FAERS, FDA Adverse Event Reporting System; ICIs, immune checkpoint inhibitors; PD-1, programmed cell death protein 1; PD-L1, programmed death-ligand 1.

### Data analysis

We conducted a comprehensive analysis of all the literature-identified cases, systematically evaluating key clinical parameters including patient demographics (age and sex), case origin, clinical outcomes, specific ICI agents (with corresponding drug classes), time to onset, presenting symptoms, therapeutic interventions, and treatment indications. Disproportionality analysis and Bayesian analysis were employed using four algorithms (reporting odds ratio (ROR), proportional reporting ratio (PRR), Bayesian confidence propagation neural network (BCPNN), and multi-item gamma Poisson shrinkage (MGPS)) to assess the associations between different classes of ICIs and HP.

ROR compares how often a specific drug is reported with HP relative to all other drugs in the database. ROR > 1 means the drug is more likely to be linked to HP than other drugs. PRR is similar to ROR but focuses on the proportion of HP reports for a drug vs other drugs. PRR ≥ 2 suggests a potential signal. BCPNN uses Bayesian statistics to calculate the information component (IC), which quantifies how much more often HP occurs with a drug than expected. IC > 0 means HP is reported more than expected. IC025 > 0 confirms a robust signal. MGPS adjusts for rare events and random noise, calculating the empirical Bayesian geometric mean (EBGM). EBGM05 > 2 signals a significant association.

These algorithms were used to identify positive signals if any algorithm met predefined threshold criteria (Table 3). Categorical data were described as frequency (percentage), while continuous data were expressed as median values. Data analysis was performed using R language (version 4.4.1) and SPSS (version 23.0).

**Table 3:** Four major algorithms used for signal detection.

Algorithms	Equation	Signal detection criteria
ROR	ROR = (a/c)/(b/d)	95% CI > 1, *n* ≥ 3
	95% CI = e^ln (ROR) ± 1.96 (1/a + 1/b + 1/c + 1/d) ^^^ 0.5^	
PRR	PRR = [a (c + d)]/[c/(a + b)]	PRR ≥ 2, *χ*^2^ ≥ 4, *n* ≥ 3
	*χ*^2^ = [(ad−bc) ^ 2] (a + b + c + d)/[(a + b) (c +2 d) (a + c) (b + d)]	
BCPNN	IC = log_2_a (a + b + c + d)/(a + c)/(a + b)	IC025 > 0
	95% CI = E (IC) ± 2V (IC) ^ 0.5	
MGPS	EBGM = a (a + b + c + d)/(a + c)/(a + b)	EBGM05 > 2
	95% CI = e^ln (EBGM) ± 1.96 (1/a + 1/b + 1/c + 1/d) ^^^ 0.5^	

Equation: a, number of reports containing both the target drug and target adverse drug reaction; b, number of reports containing other adverse drug reactions of the target drug; c, number of reports containing the target adverse drug reaction of other drugs; d, number of reports containing other drugs and other adverse drug reactions. 95% CI, 95% confidence interval; *n*, the number of reports; *χ*^2^, chi-squared; IC, information component; IC025, the lower limit of 95% CI of the IC; E (IC), the IC expectations; V (IC), the variance of IC; ROR, reporting odds ratio; PRR, proportional reporting ratio; BCPNN, Bayesian confidence propagation neural network; MGPS, multi-item gamma Poisson shrinker; EBGM, empirical Bayesian geometric mean; EBGM05, the lower limit of 95% CI of EBGM.

## Results

### Clinical profiles of nine HP cases

Our systematic review identified nine documented cases of ICI-induced hypoparathyroidism (HP) in the literature (4, 5, 6, 7, 8, 9, 10, 11, 12) (Tables 4 and 5). The cohort demonstrated distinct clinical and demographic characteristics: treatment regimens consisted of PD-1 inhibitors (four cases, 44.4%), CTLA-4/PD-1 inhibitor combinations (four cases, 44.4%), and PD-L1 inhibitors (one case, 11.1%). Patients were predominantly male (88.9%), with a median onset age of 70 years (range 53–76 years), and underlying malignancies were distributed between malignant melanoma (44.4%) and lung cancer (55.6%).

**Table 4:** Basic data with hypoparathyroidism caused by ICIs.

References	Gender	Age (year)	Country	Indication	Medication	Target for drugs	Dose	Duration (days)	Complaints	Treatment	Outcome
Win *et al.* (4)	M	73	US	Malignant melanoma	Ipilimumab + nivolumab	CTLA-4 + PD-1	NR	45	Imbalance, general weakness, abdominal pain	Calcium gluconate, ergocalciferol, calcium carbonate, calcitriol	Parathyroid gland function did not recover
Umeguchi *et al.* (5)	M	64	JP	Non-small cell lung cancer	Pembrolizumab	PD-1	200 mg	21	Fatigue	Calcium gluconate, calcitriol	Calcium level normal
Trinh *et al.* (6)	M	53	CH	Malignant melanoma	Ipilimumab + nivolumab	CTLA-4 + PD-1	3 mg kg^−1^ + 1 mg kg^−1^	28	Generalized paresthesia, stiffness in both hands, obstruction in throat, dizziness	Calcium gluconate, magnesium sulfate, calcitriol, calcium carbonate	PTH and calcium level remained low
Piranavan *et al.* (7)	F	61	US	Small cell lung cancer	Nivolumab	PD-1	NR	120	Nausea, vomiting, epigastric pain, constipation, generalized weakness, bilateral distal lower limbs paresthesias	Calcium gluconate, magnesium sulfate, calcium carbonate, calcitriol	Calcium level normal, PTH remained low
Kawkgi *et al.* (8)	M	76	US	Malignant melanoma	Ipilimumab + nivolumab	CTLA-4 + PD-1	NR	210	NR	Calcium gluconate, calcium carbonate, calcitriol, hydrocortisone	Calcium level normal, PTH remained low
Dadu *et al.* (9)	M	73	US	Malignant melanoma	Ipilimumab + nivolumab	CTLA-4 + PD-1	3 mg kg^−1^ + 1 mg kg^−1^	28	Fatigue, dizziness, ataxia, abdominal bloating, slowing of speech, change in facial expression, perioral numbness, paresthesia in both hands and feet	Calcium gluconate, calcium carbonate, calcitriol, ergocalciferol	Calcium level normal, PTH remained low
Lupi *et al.* (10)	M	59	IT	Lung adenocarcinoma	Pembrolizumab	PD-1	2 mg kg^−1^	510	Confusion, drowsiness, muscle weakness, cramps	Calcium gluconate, calcium carbonate, calcitriol	Calcium level remained low
Mahmood *et al.* (11)	M	71	US	Lung adenocarcinoma	Pembrolizumab	PD-1	200 mg	45	Fatigue, weakness	Calcium carbonate, calcitriol, calcium gluconate	Calcium level normal
Kreze *et al.* (12)	M	70	CZ	Non-small cell lung cancer	Durvalumab	PD-L1	1,500 mg	76	Nausea, muscle pain with fasciculations, carpopedal spams	Calcitriol calcium gluconate, calcitriol	Calcium level normal

CTLA-4, cytotoxic T-lymphocyte-associated protein 4; ICIs, immune checkpoint inhibitors; PD-1, programmed cell death protein 1; PD-L1, programmed death-ligand 1.

**Table 5:** Examination data of hypoparathyroidism caused by ICIs.

References	Calcium level (mg·dL^−1^)	Ca^2+^ (mmol·L^−1^)	CaSR antibodies	P (mg·dL^−1^)	Mg^2+^ (mg·dL^−1^)	PTH (pg·mL^−1^)	25-hydroxyvitamin D3 (ng·mL^−1^)	Electrocardiography
Win *et al.* (4)	5.00	0.67	No described	6.60	1.50	<1.0	18.00	Right bundle branch block, QT interval prolonged
Umeguchi *et al.* (5)	6.50	NR	No described	6.20	NR	8.00	Normal	NR
Trinh *et al.* (6)	5.41	0.70	+	5.42	1.68	7.20	48.52	QT interval prolonged
Piranavan *et al.* (7)	5.80	0.85	+	4.30	1.40	7.77	23.80	QT interval prolonged
Kawkgi *et al.* (8)	5.70	0.75	-	5.10	1.70	<6.0	Normal	QT interval prolonged
Dadu *et al.* (9)	5.00	0.67	+	6.60	1.50	<1.0	18.00	No described
Lupi *et al.* (10)	6.20	0.80	+	3.70	2.20	18.00	12.00	No described
Mahmood *et al.* (11)	6.50	NR	No described	NR	NR	4.30	21.00	No described
Kreze *et al.* (12)	5.60	0.65	No described	6.53	2.52	<5.50	15.44	No described

CaSR, calcium-sensing receptor; ICIs, immune checkpoint inhibitors; NR, not reported; PTH, parathyroid hormone.

The median latency period from ICI initiation to HP presentation was 45 days (range 21–510 days). Clinical manifestations were notable for neuromuscular involvement in the majority of cases (weakness, paresthesias, spasms, ataxia, or sensory disturbances), with only two cases reporting fatigue/weakness as primary symptoms. Biochemical profiling revealed universal hypocalcemia and hypoparathyroidism (reduced serum calcium and PTH levels), accompanied by decreased 25-hydroxyvitamin D3 (seven cases) and hyperphosphatemia (six cases). Additional findings included prolonged QT intervals (four cases) and CaSR autoantibody positivity (four cases), with three antibody-positive cases demonstrating concurrent hypomagnesemia.

Therapeutic management primarily involved calcium replacement therapy (intravenous calcium gluconate, oral calcium carbonate, and calcitriol), while corticosteroids were administered in only one instance. Notably, 66.7% of cases (6/9) exhibited persistent parathyroid dysfunction despite treatment interventions.

### Descriptive analysis of FAERS data

**Figure 1:**
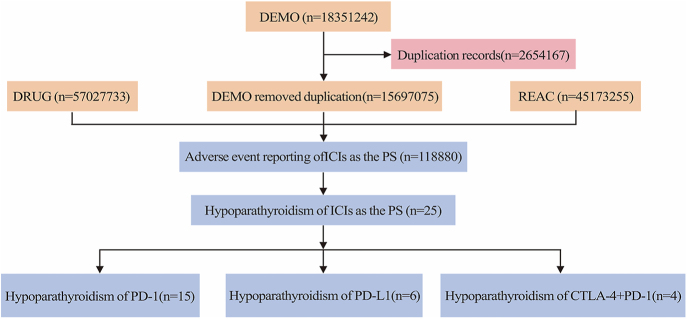
Flow chart.

Our analysis of FAERS data from Q2 2011 to Q3 2024 identified 15,697,075 unique adverse event reports after deduplication, of which 118,880 (0.76%) were ICI-related. Within this ICI-associated subset, we identified 25 cases (0.02%) of hypoparathyroidism (HP). The distribution by ICI class revealed: PD-1 inhibitors (15 cases, 60%), PD-L1 inhibitors (six cases, 24%), and CTLA-4/PD-1 inhibitor combinations (four cases, 16%) (Fig. 1).

**Figure 2:**
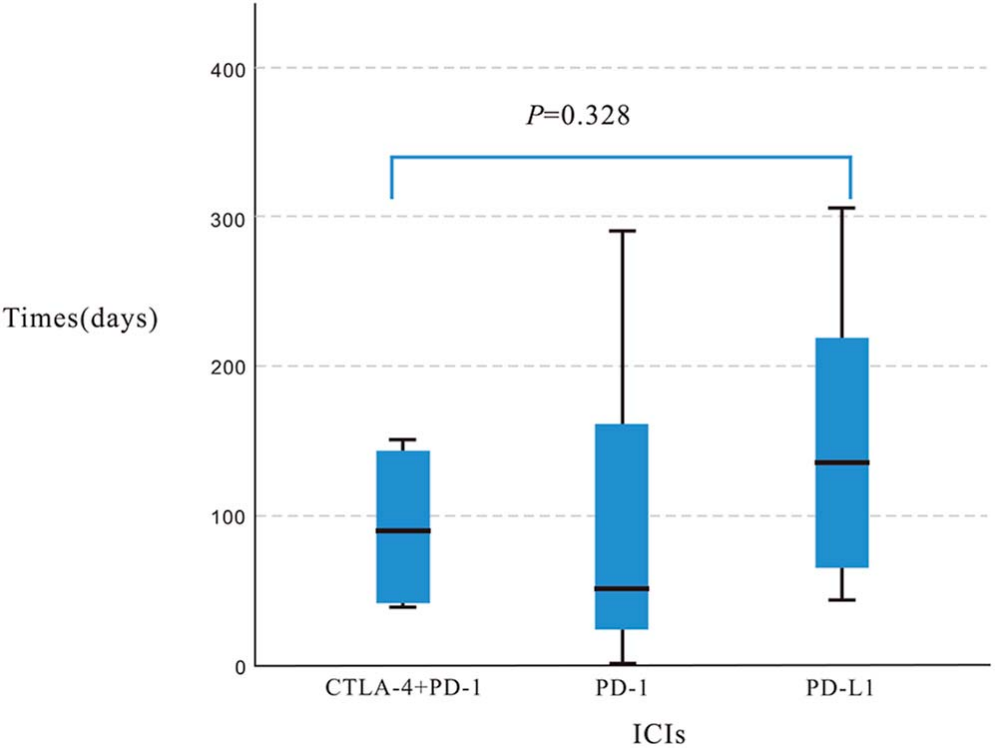
Comparison of development time of hypoparathyroidism caused by ICIs.

Lung cancer represented the most frequent indication (36%) among HP cases. The overall median time-to-onset was 64 days (range: 1–305 days), with class-specific variations observed: PD-1 inhibitors (median 50 days, range 1–292), PD-L1 inhibitors (median 135 days, range 43–305), and combination therapy (median 89 days, range 37–150). However, these differences did not reach statistical significance (*P* = 0.328, Kruskal–Wallis test) (Fig. 2).

**Figure 3:**
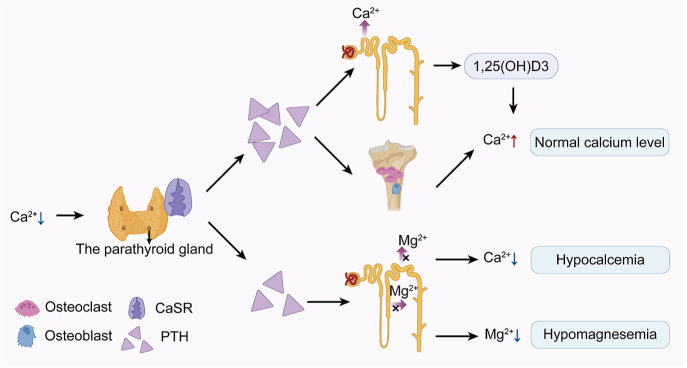
The pathophysiological mechanisms of HP.

### Association between different ICI categories and hypoparathyroidism

Our pharmacovigilance analysis revealed significant safety signals for all ICI classes in relation to HP development, with particularly robust associations observed for CTLA-4/PD-1 inhibitor combination therapy (Table 6). The combination regimen demonstrated the strongest safety signals across all four analytical algorithms, as evidenced by an IC of 2.59 (IC025 = 1.29), a ROR of 6.04 (95% CI: 2.26–16.13), a PRR of 6.04 (*χ*^2^ = 16.71), and an EBGM of 6.01 (EBGM05 = 2.64).

**Table 6:** Associations of ICIs with hypoparathyroidism.

Drugs	Number	ROR (95%CI)	PRR (*χ*^2^)	EBGM (EBGM05)	IC (IC025)
ICIs	25	5.35 (3.59–7.98)	5.35 (85.44)	5.2 (3.73)	2.38 (1.8)
PD-1	15	5.20 (3.12–8.67)	5.2 (49.77)	5.11 (3.33)	2.35 (1.62)
PD-L1	6	5.02 (2.25–11.21)	5.02 (19.14)	4.98 (2.54)	2.32 (1.22)
CTLA-4 + PD-1	4	6.04 (2.26–16.13)	6.04 (16.71)	6.01 (2.64)	2.59 (1.29)

95% CI, 95% confidence interval; *χ*^2^, chi-squared; IC, information component; IC025, the lower limit of 95% CI of the IC; ROR, reporting odds ratio; PRR, proportional reporting ratio; EBGM, empirical Bayesian geometric mean; EBGM05, the lower limit of 95% CI of EBGM; CTLA-4, cytotoxic T-lymphocyte-associated protein 4; ICIs, immune checkpoint inhibitors; PD-1, programmed cell death protein 1; PD-L1, programmed death-ligand 1.

## Discussion

The parathyroid glands, as critical components of the human endocrine system, serve as the primary source of parathyroid hormone (PTH) – a key regulator of calcium-phosphate homeostasis. HP represents a clinical syndrome resulting from either deficient PTH secretion or impaired hormonal action, manifesting as a characteristic triad of hypocalcemia, hyperphosphatemia, and consequent clinical sequelae, including soft tissue calcification and heightened neuromuscular excitability (13, 14). The clinical presentation exhibits considerable variability, contingent upon both the severity of calcium deficiency and individual patient factors. Our pharmacovigilance study and literature review demonstrate that ICI-induced HP predominantly manifests with neuromuscular symptomatology, with the most frequently reported symptoms including muscular weakness, peripheral paresthesias, carpopedal spasms, ataxic movements, and various sensory disturbances, reflecting the crucial role of calcium in neuronal and muscular membrane stability.

HP remains an uncommon clinical entity, with established etiologies primarily including post-surgical complications, autoimmune destruction, genetic disorders (e.g., DiGeorge syndrome), granulomatous infiltration, and radiation-induced damage (15). The novel association between ICIs and HP was first documented in 2017 in a patient receiving combined ipilimumab (CTLA-4 inhibitor) and nivolumab (PD-1 inhibitor) therapy (4). Our comprehensive analysis reveals distinct patterns in ICI-associated HP: literature-reported cases showed equal distribution between PD-1 inhibitor monotherapy and CTLA-4/PD-1 combination therapy (44.4% each), whereas FAERS data demonstrated a predominance of PD-1 inhibitor-associated cases (60.0%), followed by PD-L1 inhibitors, with no reported cases for CTLA-4 inhibitor monotherapy. Importantly, pharmacovigilance analysis identified the strongest HP association for CTLA-4/PD-1 combination therapy, while PD-1 and PD-L1 inhibitors exhibited comparable risk profiles.

These findings align with the established spectrum of ICI-related endocrinopathies, where combination therapy demonstrates the highest overall incidence of endocrine adverse events (16). The pattern of specific endocrine toxicities varies considerably: pituitary inflammation shows predilection for CTLA-4 inhibition (occurring in ∼4% of ipilimumab-treated patients) (17), while thyroid dysfunction predominantly accompanies PD-1 blockade and combination therapy (18). Notably, autoimmune diabetes exhibits near–exclusive association with PD-1/PD-L1 inhibitors (either as monotherapy or in combination), with minimal occurrence following CTLA-4 inhibitor monotherapy (19), highlighting the distinct immunological mechanisms underlying different ICI-associated endocrinopathies.

Our analysis revealed a significant gender disparity in ICI-induced HP, with male predominance observed in both case reports (88.9%) and FAERS data (72.0%). This finding aligns with previous research by Zhai *et al.*, demonstrating increased susceptibility of male patients to drug-induced endocrine toxicities (20), suggesting the need for enhanced monitoring in this population. The temporal patterns of ICI-related endocrine toxicities exhibit class-specific characteristics: while PD-1 inhibitor-associated endocrinopathies typically emerge between weeks 10–24, CTLA-4 inhibitor-induced hypophysitis may manifest earlier (weeks 7–8). Combination therapy accelerates toxicity onset (median ∼12 weeks). In our cohort, HP demonstrated comparable timing to hypophysitis, with median onset of 45 days (range 21–510) in the literature cases and 64 days (range 1–305) in FAERS reports. Although we observed a trend toward earlier onset with PD-1 inhibitors (median 50 days) versus combination therapy (median 89 days) and PD-L1 inhibitors (median 135 days), these differences lacked statistical significance (*P* = 0.328), possibly due to limited sample size – warranting validation in larger studies.

Notably, four HP cases exhibited prolonged QT intervals, likely attributable to hypocalcemia-induced electrophysiological alterations. The pathophysiology involves: i) diminished calcium-mediated sodium channel blockade, increasing phase 0 depolarization and phase 4 repolarization rates; ii) consequent enhancement of myocardial conduction velocity and automaticity; and iii) characteristic ECG changes, including ST-segment/QT prolongation and T-wave abnormalities in mild cases, progressing to premature contractions or tachyarrhythmias in severe hypocalcemia. These findings underscore the importance of cardiac monitoring in HP management.

The discovery of calcium-sensing receptor (CaSR) autoantibodies has revolutionized our understanding of HP pathogenesis. Building upon Li *et al.*’s seminal 1996 discovery of CaSR autoantibodies in HP patients (21) and Kifor *et al.*’s 2004 confirmation of their distinct pathological role (22), our current understanding reveals CaSR as a multifaceted G protein-coupled receptor that integrates calcium homeostasis with broader physiological processes. Through its interactions with divalent cations (Ca^2+^, Mg^2+^), and amino acids, CaSR modulates multiple signaling pathways, including phospholipase C/A2/D, adenylate cyclase inhibition, and MAPK activation, thereby influencing critical cellular processes from gene expression to differentiation (23) (Fig. 3).

Our study provides clinical evidence supporting this molecular paradigm, having identified CaSR autoantibodies in ICI-induced HP cases. These antibodies likely disrupt calcium homeostasis through dual mechanisms: i) suppressing parathyroid cell proliferation, and ii) impairing PTH synthesis/secretion, resulting in the characteristic biochemical triad of hypocalcemia, hyperphosphatemia, and inappropriately low PTH levels. Notably, the coexistence of hypomagnesemia in 75% of our CaSR-positive cases (3/4 patients) aligns with Kinoshita *et al.*’s demonstration of CaSR-mediated magnesium wasting in the nephron’s thick ascending limb and distal convoluted tubule (24).

Among the nine identified cases, six patients exhibited persistently low parathyroid hormone (PTH) levels despite therapeutic interventions. In contrast to other immune-related adverse events (irAEs), endocrine immune-related adverse events (eirAEs) frequently necessitate lifelong management. The standard therapeutic approach for HP involves calcium supplementation combined with vitamin D analogs to maintain serum calcium and phosphate concentrations within the target range. As recommended by the Japan Endocrine Society, acute hypocalcemia should be managed with intravenous administration of 8.5% calcium gluconate (10–20 mL infused over 10–20 min, followed by continuous infusion at 2–4 mL/h) (25). For non-urgent cases, oral active vitamin D supplementation (e.g., alfacalcidol, 1–3 μg/day) is indicated. During chronic management, the dosage of active vitamin D should be titrated to maintain serum calcium levels within the range of 7.5–8.5 mg/dL, while ensuring a urinary calcium-to-creatinine ratio below 0.3.

This study provides the first comprehensive analysis of ICI-induced HP using both case reports and pharmacovigilance data. Strengths include the use of four validated signal detection algorithms, which consistently identified strong associations, particularly for CTLA-4/PD-1 combination therapy. We also characterized key clinical features, including neuromuscular symptoms and CaSR autoantibodies. However, limitations include the small sample size (nine literature cases, 25 FAERS reports), which reduces statistical power and may obscure true risk differences between ICI classes. In addition, FAERS data are subject to underreporting and reporting bias, potentially overestimating severity. Despite these limitations, our findings highlight HP as a rare but important toxicity requiring calcium and PTH monitoring, especially with combination regimens. Larger prospective studies are needed to confirm these observations and guide management.

## Conclusion

Our analysis of the FAERS database revealed significant signals of HP associated with all three major classes of ICIs, with the highest risk observed in patients receiving combined CTLA-4 and PD-1 inhibitor therapy. These findings underscore the importance of heightened clinical vigilance for endocrine toxicity during ICI treatment. To optimize patient management, further research, including pharmacovigilance studies, large-scale cohort analyses, and prospective clinical trials, is essential to establish evidence-based therapeutic strategies for ICI-induced HP.

## Declaration of interest

The authors have no relevant affiliations or financial involvement with any organization or entity with a financial interest in, or financial conflict with, the subject matter or materials discussed in the manuscript. This includes employment, consultancies, honoraria, stock ownership or options, expert testimony, grants or patents received or pending, or royalties.

## Funding

This study was supported by Medical Science Research Project of Hebei (No. 20220467). 
